# Leptin is required for hypothalamic regulation of miRNAs targeting POMC 3′UTR

**DOI:** 10.3389/fncel.2015.00172

**Published:** 2015-05-06

**Authors:** Adel Derghal, Mehdi Djelloul, Coraline Airault, Clément Pierre, Michel Dallaporta, Jean-Denis Troadec, Vanessa Tillement, Catherine Tardivel, Bruno Bariohay, Jérôme Trouslard, Lourdes Mounien

**Affiliations:** ^1^Faculté des Sciences, Aix Marseille Université, PPSN EA 4674Marseille, France; ^2^Stem Cell Laboratory for CNS Disease Modeling, Department of Experimental Medical Science, Wallenberg Neuroscience Centre, Lund Stem Cell Center, Lund UniversityLund, Sweden; ^3^Biomeostasis, Nutritional Behavior and Metabolic DisordersMarseille, France

**Keywords:** microRNA, melanocortin, hypothalamus, leptin, food intake

## Abstract

The central nervous system (CNS) monitors modifications in metabolic parameters or hormone levels and elicits adaptive responses such as food intake regulation. Particularly, within the hypothalamus, leptin modulates the activity of pro-opiomelanocortin (POMC) neurons which are critical regulators of energy balance. Consistent with a pivotal role of the melanocortin system in the control of energy homeostasis, disruption of the *POMC* gene causes hyperphagia and obesity. MicroRNAs (miRNAs) are short noncoding RNA molecules that post-transcriptionally repress the expression of genes by binding to 3′-untranslated regions (3′UTR) of the target mRNAs. However, little is known regarding the role of miRNAs that target POMC 3′UTR in the central control energy homeostasis. Particularly, their interaction with the leptin signaling pathway remain unclear. First, we used common prediction programs to search for potential miRNAs target sites on 3′UTR of POMC mRNA. This screening identified a set of conserved miRNAs seed sequences for *mir-383*, *mir-384-3p*, and *mir-488*. We observed that *mir-383*, *mir-384-3p*, and *mir-488* are up-regulated in the hypothalamus of leptin deficient ob/ob mice. In accordance with these observations, we also showed that *mir-383*, *mir-384-3p*, and *mir-488* were increased in db/db mice that exhibit a non-functional leptin receptor. The intraperitoneal injection of leptin down-regulated the expression of these miRNAs of interest in the hypothalamus of ob/ob mice showing the involvement of leptin in the expression of *mir-383*, *mir-384-3p*, and *mir-488*. Finally, the evaluation of responsivity to intracerebroventricular administration of leptin exhibited that a chronic treatment with leptin decreased *mir-488* expression in hypothalamus of C57BL/6 mice. In summary, these results suggest that leptin modulates the expression of miRNAs that target POMC mRNA in hypothalamus.

## Introduction

The control of energy homeostasis is finely tuned by endocrine and neural mechanisms that cooperate to maintain the balance between caloric intake and energy expenditure. In this respect, the central nervous system (CNS) continuously monitors modifications in metabolic parameters (blood glucose) and/or hormones (insulin or leptin) and elicits adaptive responses such as food intake regulation and autonomic nervous system modulation (Cowley et al., 2001; Ibrahim et al., 2003; Plum et al., 2006; Mounien et al., 2010). It is now clearly established that specific neuronal networks of the hypothalamus play a pivotal role in energy homeostasis regulation (Morton et al., 2006). For instance, within the arcuate nucleus of the hypothalamus, pro-opiomelanocortin (POMC) neurons are critical regulators of energy balance and glucose homeostasis (Porte et al., 2002; Mounien et al., 2005, 2009, 2010; Parton et al., 2007; Hill et al., 2010). In accordance with this aspect, it has been shown that the disruption of the POMC and melanocortin receptor 4 (MC4R) genes in mice models causes obesity (Huszar et al., 1997; Yaswen et al., 1999), while MC3R gene-deficient mice have normal food consumption but accumulate fat (Chen et al., 2000). In humans, obesity can result from genetic deficiencies which produce a lack in the leptin receptor, POMC, or MC3/4R (Lee, 2009).

One important goal of current research is to identify the molecular mechanisms involved in the control of the expression of genes that are important to maintain energy homeostasis. It has long been acknowledged that the leptin acts as a key regulator of hypothalamic genes expression via different signaling cascades (Morton et al., 2006). For instance, when the leptin binds to the extracellular domain of its receptor (LepR), it recruits and activates the Janus kinase (JAK). JAK binds to and phosphorylates LepR at the same time. This mechanism activates signal transducer and activator of transcription 3 (STAT3). Once phosphorylated, STAT3 binds to POMC promoters, stimulating POMC expression (Morton et al., 2006). Interestingly, mice with genetic inactivation of STAT3 gain body weight (Gao et al., 2004). Altogether, the above collected data strongly suggest that the stability of energy homeostasis during environmental variation requires metabolic adjustements that are achieved through a fine regulation of genes's expression.

Because microRNAs (miRNAs) have been depicted to be another layer of gene regulation, it is not surprising that they are also involved in leptin-regulated gene expression. The miRNAs are endogenous, single-stranded, small, ~22-nucleotides noncoding RNAs, and are generally regarded as negative regulators of gene expression because they inhibe translation and/or promot mRNA degradation by base pairing to complementary sequences within the 3′untranslated region (3′UTR) of protein-coding mRNA transcripts (Bagga et al., 2005). Several studies identified miRNAs that are differentially expressed in the liver, pancreas, and adipose tissue of leptin-deficient (ob/ob) or leptin receptor-deficient (db/db) mice compared to the control animals (Lovis et al., 2008; Li et al., 2009; Nakanishi et al., 2009; Xie et al., 2009). Among these miRNAs, it has been shown that the pancreatic expression levels of *mir-375* are aberrant in ob/ob mice, indicating that they contribute to insulin resistance in this model (Poy et al., 2009). Additional studies provide evidence for the involvement of *mir-335* in lipid metabolism of the liver and the adipose tissue of ob/ob and db/db mice (Nakanishi et al., 2009). In the context of CNS, it has been shown that *mir-200a*, *mir-200b*, and *mir-429* are up-regulated in the hypothalamus of ob/ob and db/db mice (Crépin et al., 2014).

Recently, it has been shown that conditional deletion of the RNAse III ribonuclease Dicer (involved in miRNAs maturation) from POMC-expressing cells results in obesity and diabetes which is associated with a neurodegenerescence of POMC neurons in the hypothalamus (Schneeberger et al., 2012; Greenman et al., 2013). These observations strongly suggest that miRNAs are important regulators of POMC neuron activity. In this context, the characterization of the miRNAs that target directly POMC mRNA and their interaction with the leptin signaling pathway remain unclear.

In the present study, we focused our attention on the specific miRNAs targeting POMC 3′UTR. Based on bioinformatic predictions of their involvement in POMC-signaling pathway and their conservation among vertebrates, the expression of *mir-383*, *mir-384-3p*, and *mir-488* were investigated in models of obesity characterized by a decrease of POMC mRNA expression and leptin insufficiency (ob/ob) or leptin insensitivity (db/db) (Mizuno et al., 1998). Then, we further analyzed the role of leptin on the expression level of these miRNAs using different models of leptin-treated mice.

## Methods

### Animals

Experiments were carried out on different types of mice: C57BL/6, ob/ob, and db/db mice were purchased from Charles River (France). Fluorescence *in situ* hybridization (FISH) experiments were performed using male POMC-Tau-Topaz GFP transgenic mice developed by Pinto et al. (2004). To assess GFP expression in POMC-Tau-Topaz GFP mice, we carried out PCR on tail genomic DNA. GFP transgene was detected using the forward primer 5′-GCCACAAGTTCAGCGTGTCC-3′ and the reverse primer 5′-GCTTCTCGTTGGGGTCTTTGC-3′, with the following PCR conditions: 5 min at 95°C, 36 cycles at 95°C for 30 s, 64°C for 30 s, and 72°C for 40 s, followed by a final step at 72°C for 7 min. The amplicon size was 573 bp.

All animals were individually housed in a pathogen-free facility at controlled temperature on a 12/12 h light/dark cycle (lights from 0700 to 1900 h) with standard pellet diet (AO4) and water available *ad libitum*. All experiments were conducted in conformity with the rules set by the EC Council Directive (2010/63/UE) and the French “Direction Départementale de la Protection des Populations des Bouches-du-Rhône” (License no. 13.435 and no. 13.430). Protocols used are in agreement with the rules set by the Comité d'Ethique de Marseille, our local Committee for Animal Care and Research. Every precaution was taken to minimize animal stress and the number of animals used.

### miRNA prediction

To search for miRNAs that might regulate mouse POMC expression, we used the following public prediction algorithms and database: Targetscan (http://www.targetscan.org/) and miRanda (http://www.microrna.org/microrna/home.do). Using these different algorithms, we selected the miRNAs that are conserved among the vertebrates (Targetscan) and that have a good mirSVR scores (miRanda).

### Surgery and injections

For the intraperitoneal injection (i.p.), ob/ob, and C57BL/6 mice were injected between 1100 and 1200 h with recombinant murine leptin (Peprotech, France) (*n* = 5, 5 mg/kg) or saline. Mice were sacrified 4 h after i.p. injection.

Intracerebroventricular (i.c.v) cannula placement and injections were performed as described previously (Girardet et al., 2011). Animals were anesthetized by an i.p. injection of ketamine (100 mg/kg; Imalgen 1000, Merial, France) and xylazine (6 mg/kg; Rompun, Bayer, France), and placed in a digital stereotaxic apparatus (Model 502600, WPI) coupled to the neurostar software (Neurostar GmbH, Germany). A 26-gauge stainless steel cannula was implanted into the lateral ventricle at the following coordinates: 0.3 mm posterior to bregma, 1.1 mm lateral to the midline, and 2.6 mm ventral to the skull surface. The cannula was secured to the skull with dental cement and sealed with removable obturators. The animals were sutured, placed in individual cages and allowed to recover for 7 days. During this recovery period, animals were injected with physiological saline every day for habituation. One week post-surgery, mice were administered either 10 μl (2 μl/min) of physiological saline or leptin (0.5 μg/μl) solution at the beginning of the dark phase. The correct cannula positioning was checked for each animal at the end of the experiment by cresyl violet staining of brain sections (Supplementary Figure 1).

The exogenous leptin was detected in the hypothalamus of ob/ob and C57BL/6 mice after i.p. and i.c.v injection by western blotting (Supplementary Figure 2 and Supplementary Material).

### Quantitative RT-PCR (qRT-PCR) analysis

Mice were killed by decapitation and the different brain regions were collected and frozen in liquid nitrogen and stored at −80°C until protein or RNA extraction. Total RNA was extracted with TRI Reagent (Sigma-Aldrich, France). For RNA quantification, cDNA was synthetized by 2 μg total RNA with M-MLV Reverse Transcriptase (Promega Corporation, WI, USA). For miRNAs quantification, cDNA was synthetized by 1 μg total RNA by the qScript microRNA Quantification System (Quanta Biosciences, MD, USA). For real-time PCR, we used LightCycler 480 (Roche, Germany). We used *Pomc* forward primer 5′-TGAACATCTTTGTCCCCAGAGA-3′ and reverse 5′-TGCAGAGGCAAACAAGATTGG-3′; β Actin forward primer 5′-GATCTGGCACCACACCTTCTACA-3′ and reverse 5′- TGGCGTGAGGGAGAGCATAG-3′; *Gapdh* forward primer 5′- TTCTCAAGCTCATTTCCTGGTATG-3′ and reverse primer 5′- GGATAGGGCCTCTCTTGCTCA-3′. PCR was initiated by one cycle of 95°C for 10 min, followed by 40 cycles of 10 s at 95°C, 30 s at 60°C, and 2 s at 72°C, followed by a holding at 40°C. For miRNAs, *U6* and *Sno202* were used as normalizers for miRNA quantification. For *U6* we used forward primer 5′-ATTGGAACGATACAGAGAAGATT-3′ and reverse primer 5′-GGAACGCTTCACGAATTTG-3′; *Sno202* forward primer 5′-CTTTTGAACCCTTTTCCATCTG-3′ *mir-383* forward primer 5′-CAGATCAGAAGGTGACTGTG-3′; *mir-384-3p* forward primer 5′- TGTAAACAATTCCTAGGCAATGA-3′; *mir-471-3p* forward primer 5′- TGAAAGGTGCCATACTATGTAT-3′, *mir-488* forward primer 5′- CCCAGATAATAGCACTCTCAA-3′; for the reverse primers we used the Universal Primer (Quanta Biosciences). We performed quantitative PCR according to the manufacturer's instructions (Quanta Biosciences).

### miRNA fluorescent *In Situ* hybridization (FISH) and GFP immunohistochemistry

For the detection of miRNAs in POMC neurons, POMC-Tau-Topaz GFP transgenic mice were perfused intracardially with heparin 10% in PBS-Diethylpyrocarbonate (DEPC)-treated (0.1% PBS, 0.1 M, pH 7.4) followed by 4% PFA in PBS-DEPC maintained at 4°C under ketamine/xylazine anesthesia (100 and 15 mg/kg, respectively). The brains were removed and postfixed for 2 h in 4% PFA/DEPC, cryoprotected for 48 h in 30% sucrose in PBS/DEPC at 4°C and frozen in O.C.T. (Tissue-Tek; Sakura Finetek, USA). Subsequently the brains were sliced at 12 μm thickness from −1.34 to −2.70 mm relative to bregma and transferred serially on poly-L-lysine and gelatin-coated Super Frost slides (Fisher, PA, USA). Slides were stored at −20°C until FISH. The miRNA FISH was based on Thompson et al. (2007) protocol. Slides were removed from storage at −20°C and air dried at 37°C for 30 min then placed in 4 % PFA in PBS-DEPC for 20 min and washed in PBS-DEPC 2 times for 10 min. Sections were treated with 10 mg/ml proteinase K for 6 min at room temperature and washed in PBS-DEPC for 10 min. Fixation with 4% PFA in PBS-DEPC was performed for 15 min and rinse in DEPC-treated water. Slides were treated with acetic anhydride (Triethanolamine, 0.1 M, pH 8.0; Acetic anhydride 1:400) 2 times for 5 min and washed with PBS-DEPC for 10 min. Sections were incubated in pre-hybridization buffer (50 % Formamide; 5× SSC; 0.3 mg/ml RNA Yeast; 100 μg/ml heparin; 1× Denhardt's solution (2% bovine serum albumin, 2% polyvinylpyrrolidone; 2% Ficoll 400); 0.1% Tween 20; 0.1% CHAPS; 5 mM EDTA; 0.3 nmole/ml DNA Random Primer 12-mer) for 2.5 h. Hybridization was performed overnight at 37°C in the same buffer with 3′end 6 Fluorescein amidite (6FAM)-labeled DNA oligonucleotide probes at 5 μg/ml. We used the antisense probes 5′-AGCCACAGTCACCTTCTGATCTTT-3′-6FAM for *mir-383*; 5′-TTACATTGCCTAGGAATTGTTTACATA-3′-6FAM for *mir-384-3p*; 5′-AAAACTCTCACGATAATAGACCCTT-3′-6FAM for *mir-488*. Scrambled probes were used for negative control for each experiment. The scramble sequences were 5′-TTCCGACAACTGCACTCTATGTTC-3′6FAM for scramble (sc) *mir-383*, 5′-CATGAATATTCCGTGGTTAATCATTTA-3′6FAM for *scmir-384-3p* and 5′-AGATTCTCAACCTGCTTTACAAAGC-3′6FAM for *scmir-488*. Slides were washed with 2× SSC for 15 min at 37°C. High-stringency tetramethyl ammonium chloride (TMAC, Acros Organics, NJ, USA) washes were performed 2 times for 5 min at 54°C and 1 time for 10 min at 54°C then rinsed in PBS-DEPC with 0.1% Triton X-100 (PBT) for 10 min. Slides were incubated in PBT with 20% horse serum for 1 h for blocking. Rabbit polyclonal antibody anti-fluorescein (1:500, Gen Tex, TX, USA) and mouse monoclonal anti-GFP (1:500, Abcam, MA, USA) antibodies in PBT with 20% horse serum were added to sections and incubated for overnight at 4°C. Slides were washed three times in PBT for 10 min. Alexa 488 fluor-conjugated goat anti-mouse and Alexa 594 fluor-conjugated donkey anti-rabbit (1:500, Life Technologies, France) with PBS 3% horse serum and 0.3% Triton X-100 were added sequentially to slides for 1.5 h at room temperature and sections were washed three times with PBS.

Sections were finally coverslipped with mounting medium for fluorescence microscope preparation. Sections were observed using a Zeiss LSM 700 confocal microscope (Zeiss, France) associated to ZEN 2012 software and a DXM 1200 Camera (Nikon, France) coupled to ACT-1 software. For quantitative analysis, cells were counted manually using the Image J analysis system (National Institutes of Health, USA). For sections stained for both eGFP and miRNA probes, the double labeled cells were examined at multiple focal levels and at appropriate magnifications to ensure that single cells were indeed immunoreactive for both eGFP and miRNA probes. Because POMC neurons constitute a heterogeneous population in relation to their sensitivity to regulatory factors (Mounien et al., 2006a,b; Williams et al., 2010), the average number of cells counted bilaterally in 8 sections at the anterior and posterior levels of the arcuate nucleus in each animal (*n* = 4) was used for statistical comparisons.

### Statistical analysis

All data are expressed as mean ± SEM. Statistical analysis was performed by an unpaired 2-tailed Mann-Whitney test. *P* < 0.05 was considered significant.

## Results

### Identification and localization of miRNAs of interest in the hypothalamus

**Figure 1:**
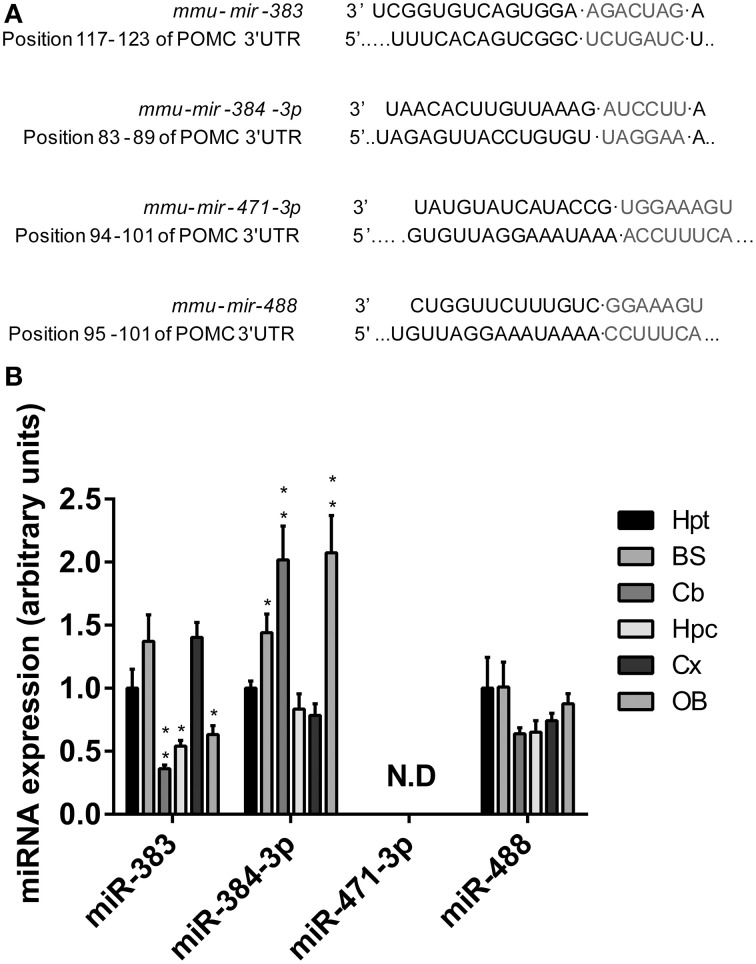
**Characterization of microRNAs identified as potential regulators of POMC mRNA expression. (A)** Predicted conserved binding sites for miRNAs were bioinformatically identified in the sequence of the POMC mRNA 3' UTR. **(B)** The four miRNAs with the highest conservation degree were quantified by qRT-PCR for their presence in the hypothalamus (Hpt), brainstem (BS), cerebellum (Cb), hippocampus (Hpc), cortex (Cx), and olfactory bulb (OB) of wild-type mice. Relative expression levels of *mir-383*, *mir-384-3p*, *mir-471-3p*, and *mir-488* are expressed in fold change of the normalized level obtained in the hypothalamus. Values represent the mean ± SEM (*n* = 7). ^*^P < 0.05, ^**^P < 0.01 vs. hypothalamus.

We used common prediction programs (http://www.targetscan.org and http://www.microrna.org) to search for potential miRNAs target sites on the 3′UTR of Pomc mRNA. In one hand, we identified three conserved miRNAS (*mir-383*, *mir-384-3p*, and *mir-488*) with targetscan.org. In the other hand, the conserved miRNAS with a good mirSVR scores identified with microrna.org are *mir-384-3p*, *mir-371-3p*, and *mir-488* (Figure 1A). The distributions of these miRNAs of interest in the mouse CNS were investigated by means of quantitative PCR.

The expression profiles of *mir-383*, *mir-384-3p*, and *mir-488* miRNAs presented many similarities, but also major differences. Thus, in the CNS, the hypothalamus, brainstem, and cortex were the three regions that contained the highest densities of *mir-383* miRNAs (Figure 1B). In other CNS structures such as the cerebellum, hippocampus, and olfactory bulb, much lower concentrations of *mir-383* miRNA were recorded (Figure 1B). The highest amounts of *mir-384-3p* miRNAs were found in the brainstem, cerebellum, and olfactory bulb (Figure 1B). Lower levels of *mir-384-3p* miRNAs were detected in the hypothalamus, hippocampus, and cortex (Figure 1B). The *mir-488* miRNAs were widely expressed throughout the CNS with the same intensity (Figure 1B). It should be noted that *mir-471-3p* miRNA expression was undetectable whatever the brain region studied (Figure 1B). Considering these expression patterns, we focused our work on *mir-383*, *mir-384-3p* as *mir-488* which are expressed in the hypothalamus.

### The miRNAs of interest are expressed in the POMC neurons of the arcuate nucleus

**Figure 2:**
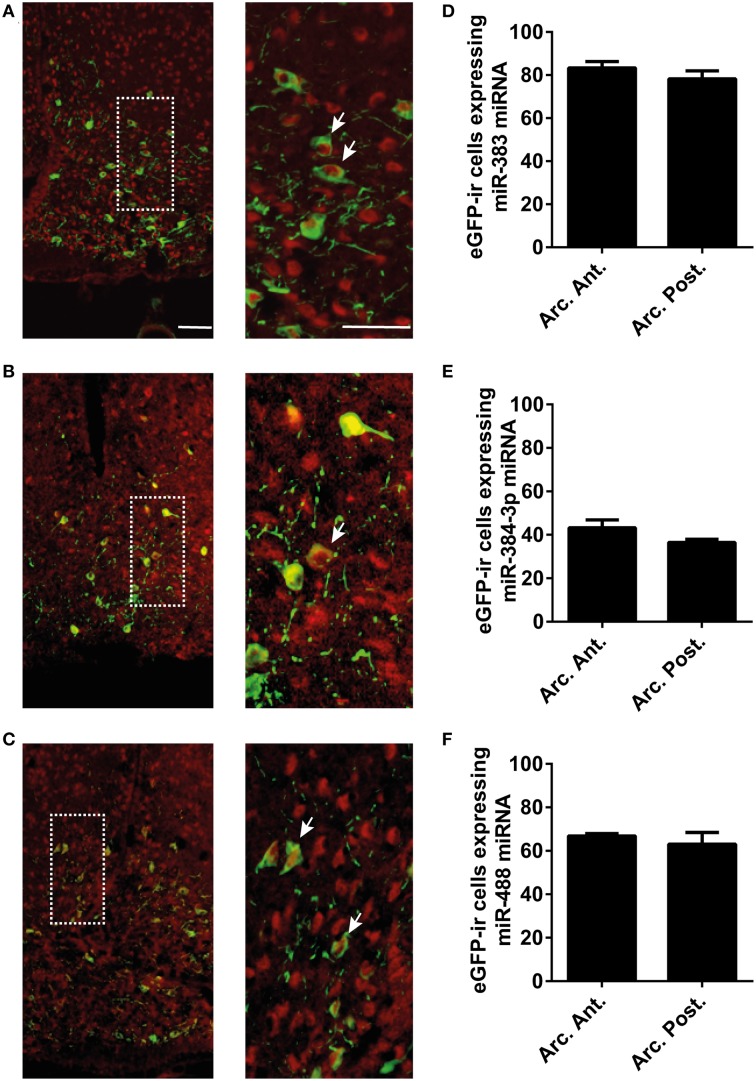
**Expression of mir-383, mir-384-3p, and mir-488 in POMC neurons of the arcuate nucleus. (A–C)** *In situ* hybridization and immunofluorescence microscopy detection of miRNAs expression in GFP positive neurons at the arcuate nucleus level of POMC-Tau-Topaz GFP mice model. **(D–F)** Quantitative analysis of GFP-positive neurons that were labeled with miRNA oligonucleotide probes at anterior (arc. ant.) and posterior (arc. post.) level of the arcuate nucleus. Scale bar = 50 μm. White arrowheads point to POMC neurons expressing miR-383, miR-384-3p or miR-488 miRNA.

In POMC-Tau-Topaz GFP mice model, double-staining of brain sections with the fluorescein-labeled *mir-383* probe and the antibody against GFP showed that a large proportion of neurons in the arcuate nucleus contained simultaneously GFP and *mir-383* miRNAs (Figure 2A). Several GFP neurons did not contain *mir-383* miRNAs and, reciprocally, several *mir-383* miRNAs-expressing neurons did not contain GFP protein (Figure 2A). Similarly, double staining of brain sections with the GFP antibody and *mir-384-3p* or *mir-488* probes showed that a large proportion of GFP neurons expressed *mir-384-3p* or *mir-488* miRNAs (Figures 2B,C). Quantitative analysis of double-labeled neurons showed that, in the whole arcuate nucleus, 80.7 ± 3.4% of the GFP-positive neurons expressed *mir-383* miRNAs, 39.8 ± 2.5% of the GFP-positive cells expressed *mir-384-3p* miRNAs and 64.9 ± 3.2% of the GFP- immunoreactive cells contained *mir-488* miRNAs (Figures 2D–F). The proportion of GFP neurons that exhibited *mir-383*, *mir-384-3p*, or *mir-488* miRNAs were not different in the anterior and posterior subdivisions of the arcuate nucleus (Figures 2D,F). The control scrambled probes did not produce any labeling (Supplementary Figure 3).

### The miRNA of interest are up-regulated in the hypothalamus of ob/ob and db/db mice models

**Figure 3:**
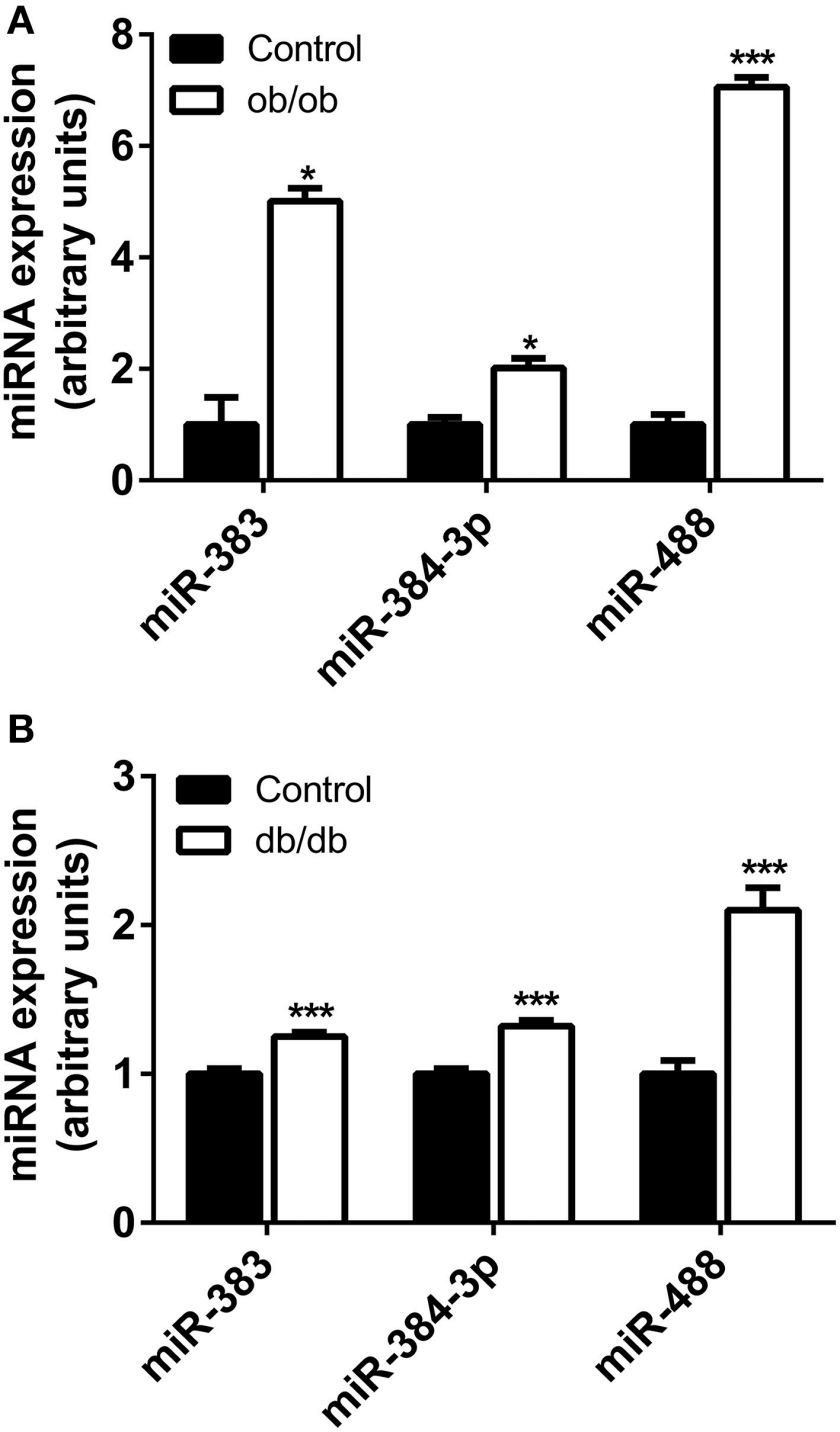
**Expression of miRNAs in the hypothalamus of ob/ob and db/db mice. (A)** Hypothalamic *mir-383*, *mir-384-3p*, and *mir-488* expression in ob/ob and control mice at 16 weeks of age. **(B)** Hypothalamic miRNAs expression in db/db and control mice at 16 weeks of age. Values represent the mean ± SEM (*n* = 5 − 10). ^*^P < 0.05, ^***^P < 0.001.

To evaluate whether leptin signaling in hypothalamus is essential for the expression of *mir-383*, *mir-384-3p*, or *mir-488* miRNAs, we analyzed the expression of these miRNAs among groups of leptin-deficient (ob/ob) mice and C57BL/6 controls. The levels of *mir-383*, *mir-384-3p*, and *mir-488* miRNAs in the hypothalamus of 16-week-old ob/ob and C57BL/6 mice were determined by qRT-PCR. Absence of leptin resulted in a significant increase in *mir-383*, *mir-384-3p, and mir-488* miRNAs (+400%, +101%; *P* < 0.05 and +605%; *P* < 0.001, respectively) (Figure 3A).

Because the lack of circulating leptin was associated with a significant increase in the miRNAs of interest, we measured the expression of *mir-383*, *mir-384-3p*, and *mir-488* miRNAs in the hypothalamus of db/db mice, a model that exhibits a non-functional leptin receptor leading to impaired leptin signaling. As shown in Figure 3B, the *mir-383*, *mir-384-3p*, and *mir-488* miRNAs levels were over-expressed in 16-week-old db/db mice compared to C57BL/6 animals (+25, +32 and +110%, respectively; *P* < 0.001).

### The expression of selected miRNAs are restored in the hypothalamus of ob/ob mice after peripheral leptin administration

**Figure 4:**
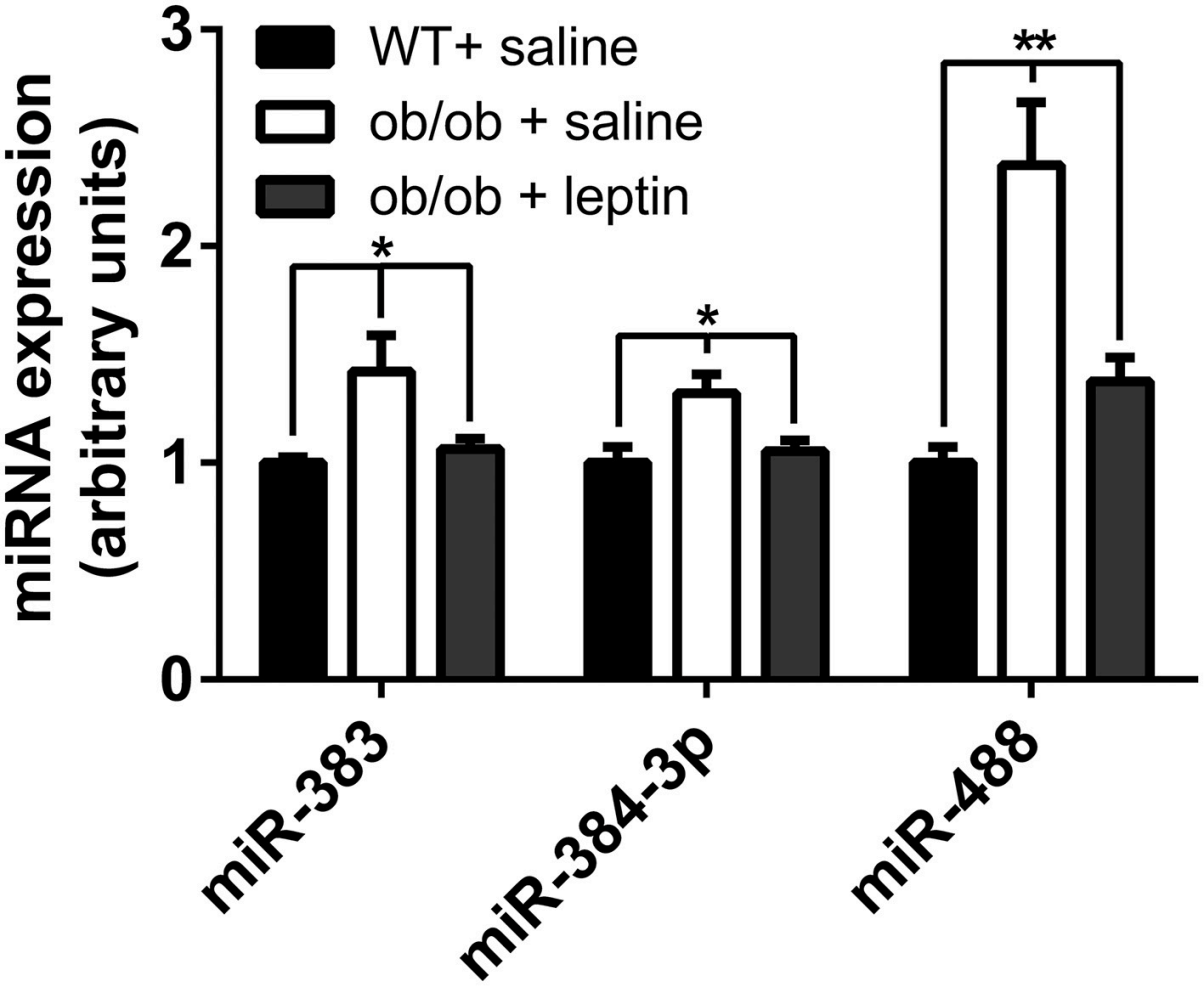
**Expression of miRNAs in the hypothalamus of peripheral leptin-treated ob/ob mice and saline-treated ob/ob and wild-type animals.** Hypothalamic miRNA expression in peripheral saline-treated animals and leptin-treated ob/ob mice at 12 weeks of age. Values represent the mean ± SEM (*n* = 5). ^*^P < 0.05, ^**^P < 0.01.

The above data suggest that leptin may be involved in the regulation of the expression of *mir-383*, *mir-384-3p*, and *mir-488* miRNAs in the hypothalamus. To directly test this hypothesis, ob/ob mice were i.p. injected with leptin at dose of 5 mg/kg and the hypothalamic expression of *mir-383*, *mir-384-3p*, and *mir-488* miRNAs was evaluated by qRT-PCR 4 h later. We observed an up-regulation of *mir-383*, *mir-384-3p*, and *mir-488* miRNAs in 12-week-old ob/ob animals treated with vehicle compared to C57BL/6 control mice (+42, +32%; *P* < 0.05 and +137%; *P* < 0.01, respectively) (Figure 4). As expected, leptin treatment significantly reduced hypothalamic expression of *mir-383*, *mir-384-3p*, and *mir-488* miRNAs in 12-week-old ob/ob animals treated with leptin compared to vehicle-treated ob/ob mice (−25, −21%; *P* < 0.05 and −58%; *P* < 0.01, respectively) (Figure 4).

### The expression of *mir*-488 is decreased in the hypothalamus of C57BL/6 mice after central leptin administration

**Figure 5:**
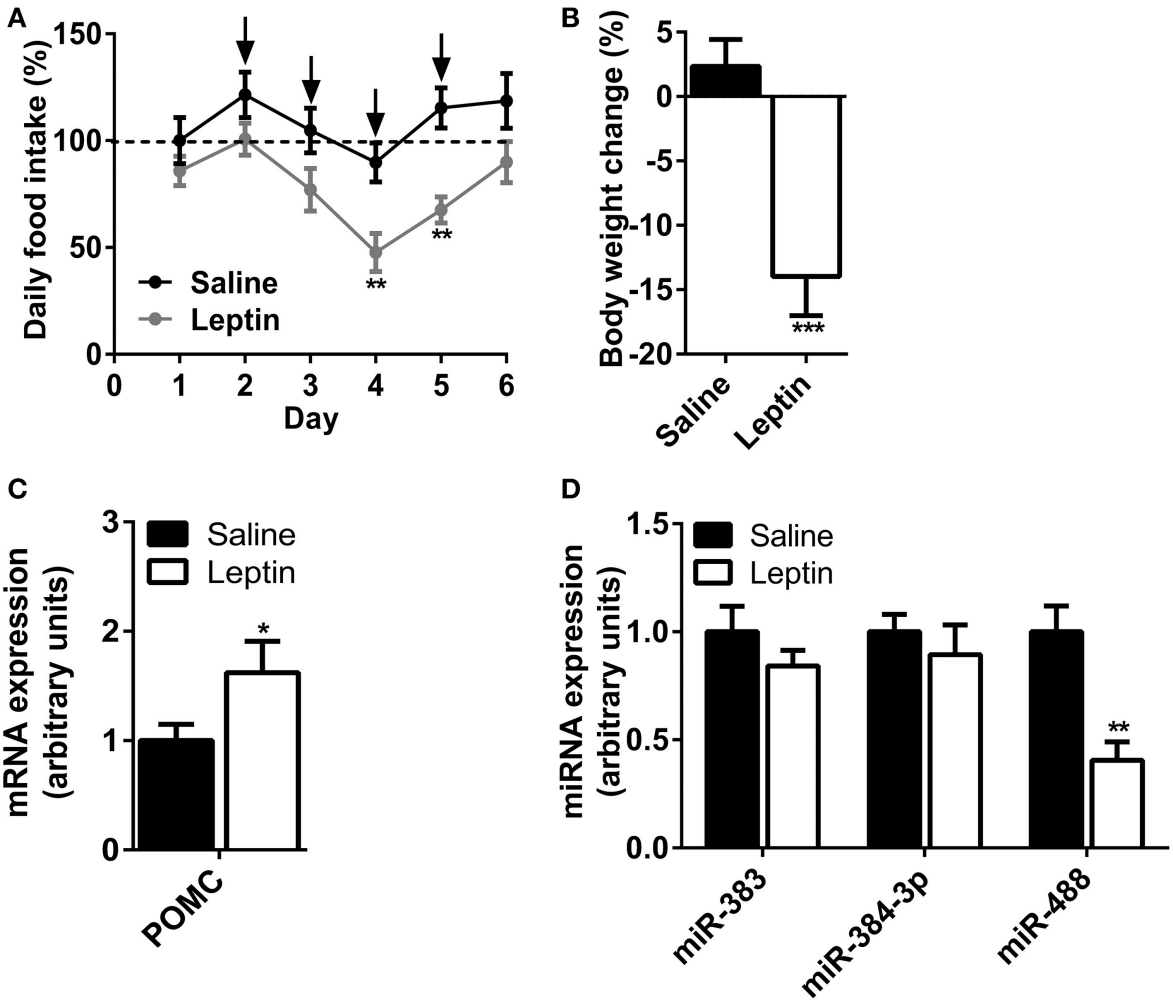
**Effect of leptin central administration on miRNA levels in the hypothalamus. (A)** Daily food intake in saline-injected and leptin-treated mice at baseline (day 1 before injection) and with daily i.c.v injections of leptin (2.5 μg; days 2–5). **(B)** Effect of leptin treatment on body weight. **(C)** Hypothalamic POMC mRNA expression in i.c.v leptin-treated mice and saline-treated animals. **(D)** Hypothalamic *mir-383*, *mir-384-3p*, and *mir-488* expression in i.c.v leptin-treated mice and saline-treated animals. Arrows indicate the injections. Values represent the mean ± SEM (*n* = 7). ^*^P < 0.05, ^**^P < 0.01, ^***^P < 0.001 vs. saline-treated animals.

Because leptin exhibits a large range of effect at peripheral level (Margetic et al., 2002), we next investigated the effect of a daily i.c.v leptin administration (5 μg/mice) during 4 days on hypothalamic expression of *mir-383*, *mir-384-3p, and mir-488* miRNAs. The sub-chronic i.c.v leptin administration significantly reduced food consumption and body weight gain in C57BL/6 mice when compared with the control animals (Figures 5A,B). The i.c.v administration of leptin significantly increased POMC mRNA level in the hypothalamus of leptin-treated group compared with the vehicle-treated animals (+62%; *P* < 0.05) (Figure 5C). Concurrently, the effects of leptin on miRNAs levels were evaluated. Chronic i.c.v administration of leptin had no effect on *mir-383* and *mir-384-3p* miRNAs levels (Figure 5D). In contrast, leptin significantly decreased *mir-488* miRNA contents in the hypothalamus after treatment (−60%; *P* < 0.01) (Figure 5D).

## Discussion

The relevance of miRNAs in the function of melanocortin pathways has been recently highlighted by the deletion of *Dicer* in POMC-expressing cells which led to a postnatal ablation of POMC neurons resulting in obesity (Schneeberger et al., 2012; Greenman et al., 2013). In this models, the absence of *Dicer* in POMC-positive neurons led to hyperphagia (Schneeberger et al., 2012) and a decrease of energy expenditure without any increase in food intake (Greenman et al., 2013). Taken together, the data suggests that miRNAs are essential for the integrity of POMC neuron activity.

Firstly, we studied the pattern of expression of miRNAs, i.e., *mir-383*, *mir-384-3p*, and *mir-488*, in the CNS. It is already established that these miRNAs are highly expressed in the brain (Chiang et al., 2010). In agreement with this data, we have found by using qRT-PCR that the highest concentrations of *mir-383* occurred in the hypothalamus, brainstem, and cortex. The highest amounts of *mir-384-3p* miRNAs were found in the brainstem, cerebellum, and olfactory bulb while *mir-488* miRNAs were widely expressed throughout the structures studied. In many brain regions, *mir-383*, *mir-384-3p*, and *mir-488* miRNAs distribution patterns did not match each other suggesting that the three miRNAs also exert specific roles. For the first time, we found that the miRNAs encoding the *mir-383*, *mir-384-3p*, and *mir-488* miRNAs are abundant in the arcuate nucleus. This indicates that a large proportion of the different cell types present in the nucleus are equipped with these miRNAs of interest. The double-staining of POMC-Tau-Topaz GFP mice brain sections with the fluorescein-labeled probes and an antibody against GFP revealed that a high proportion of POMC neurons express *mir-383*, *mir-384-3p*, and *mir-488*, suggesting that these miRNAs target the expression of protein-coding genes that are distributed in this neuronal population. Although *mir-383*, *mir-384-3p*, and *mir-488* miRNAs were evenly distributed along the rostro-caudal axis of the arcuate nucleus, the proportion of double labeled GFP/*mir-383*, GFP/*mir-384-3p*, and GFP/*mir-488* neurons did not reflect each other in terms of the proportion of their distribution. These observations are consistent with previous reports demonstrating that POMC neurons constitute an heterogeneous population in relation to their sensitivity to regulatory factors (Mounien et al., 2006a,b; Williams et al., 2010).

In the present context, of special interest is the fact that the POMC neurons are essential for the integration of peripheral signals such as hormones (leptin and insulin) and/or nutrients (glucose) (Morton et al., 2006). For instance, it has been shown recently that leptin directly acts through POMC neurons to stimulate energy expenditure (Berglund et al., 2012). One important goal of current research is to clarify the molecular mechanism involved in the integration of these multiple peripheral metabolic signals such as leptin. One path to reach this goal is to identify the intracellular mediators that allow these POMC neurons to respond to energy status modifications. Interestingly, we observed that the miRNAs targeting POMC mRNAs are up-regulated in ob/ob and db/db mice models. This data provides the first evidence that the expressions of *mir-383*, *mir-384-3p, and mir-488* are associated with an impaired leptin signaling pathway. However, the miRNAs that target POMC 3′UTR may modulate the melanocortin system at other levels notably by regulating the expression and/or activity of the prohormone convertases PC1 and PC2 which are required for the processing of POMC and the formation of α-melanocyte-stimulating hormone (Smith and Funder, 1988). In accordance with this hypothesis, we observed in the databases that *mir-488* can also target PC1 3′UTR. In accordance with this hypothesis, PC1 gene expression is different in the arcuate nucleus of leptin-treated obese ob/ob mice (Nilaweera et al., 2003). It is also conceivable that miRNAs may control the biosynthesis of agouti-related protein; the endogenous antagonist of melanocortin receptors MC3R and MC4R (Ollmann et al., 1997).

Several miRNAs have been reported to be differentially expressed in the hypothalamus of ob/ob and db/db mice, but their precise role in the regulation of the hypothalamus function remains to be determined. Recently, it has been shown that *mir-200a*, *mir-200b*, and *mir-429* are up-regulated in the hypothalamus of ob/ob and db/db mice (Crépin et al., 2014). The same group showed that daily injection of a pegylated leptin antagonist predisposed rats to obesity, promoted leptin resistance and modified the hypothalamic miRNA expression profile (Benoit et al., 2013). In our study, we identified the presence of new miRNAs that are expressed differently in the hypothalamus of ob/ob and db/db animals compared to control mice. Additional studies will be necessary to understand the regulation of these different miRNAs in the hypothalamus during the progressive onset of the obese phenotype and to determine their complex role in the regulation of feeding behavior.

We observed that the fold change modification of the selected miRNAs in the ob/ob mice increased in an age dependent way. These results suggest that age positively regulates miRNAs expression in ob/ob mice. In accordance with this hypothesis, it has been also shown that *mir-184* is differentially expressed in the pancreatic islets of ob/ob mice from age 4–16 weeks (Tattikota et al., 2014). The present study shows that leptin peripheral treatment rescues the expression of *mir-383*, *mir-384-3p*, and *mir-488* in ob/ob mice. Thus, together with the altered expression of these miRNAs in ob/ob and db/db mice, these results support the idea that leptin plays an important role in the expression of the miRNAs that target POMC mRNA in the hypothalamus. However, leptin exhibits a wide range of effects at peripheral level (Margetic et al., 2002) and its effect on miRNAs expression in the hypothalamus could thus be indirect. In order to test the effect at central level, we then performed chronic i.c.v infusion of leptin. Surprisingly, i.c.v administration of leptin in mice only decreased the expression of *mir-488* in the hypothalamus suggesting that the expression of *mir-488* is under the control of leptin. The absence of effect by central infusion of leptin on the hypothalamic expression of *mir-383* and *mir-384-3p* can be explained by the peripheral effect of leptin. For instance, it has been shown that leptin increases insulin secretion which in turn modulates the POMC neurons activity and may modulate miRNA expression (Ceddia et al., 1999; Hill et al., 2010).

Interestingly, bioinformatics prediction analysis indicates that the miRNAs targeting POMC can also target genes involved in the leptin signaling pathways such as STAT3 as well as the leptin receptor. For instance, *mir-488* could modulate the expression of STAT3. These observations suggest that, *mir-383*, *mir-384-3p*, and *mir-488* in the arcuate nucleus are involved in a complex network controlling the sensitivity of POMC neurons to peripheral signals. Additional *in vitro* experiments could clarify this last point. Recently, numerous studies identified several miRNAs as important for the hypothalamic regulation of energy homeostasis. Herzer et al. identified *mir-7a* as a hypothalamic-enriched miRNA with a high expression in Neuropeptide Y/Agouti-related orexigenic peptide neurons (Herzer et al., 2012). Interestingly, two extreme conditions of nutritional stress, i.e., caloric restriction and high fat diet-induced obesity, modified the hypothalamic pattern of expression of a set of miRNAs including *let7a*, mir-9^*^, *mir-30e*, *mir-132*, *mir-145*, *mir-200a*, and *mir-218* (Sangiao-Alvarellos et al., 2014). Taken altogether, these different studies point to the importance of miRNAs as regulators and sensors of central energy homeostasis.

In conclusion, we observed that the impairment of leptin synthesis or signaling, induced a defect in the hypothalamic expression of a subset of miRNAs that target POMC 3′UTR. It was observed that *mir-383*, *mir-384-3p*, and *mir-488* were up-regulated in ob/ob and db/db mice and *mir*-488 was down-regulated in leptin-treated mice. This indicates that the miRNAs that target POMC important mediators of leptin action in the hypothalamus. New miRNAs modulated by leptin could open important therapeutic perspectives for controlling metabolic disorders.

## Author contributions

LM planned the project. AD, MD, CA, CP, MD, VT, and CT performed experiments. LM wrote the manuscript. JT, BB, and JT helped with data evaluation, interpretation, and manuscript preparation.

### Conflict of interest statement

The authors declare that the research was conducted in the absence of any commercial or financial relationships that could be construed as a potential conflict of interest.
