# Supplementary material for: Leptin is required for hypothalamic regulation of miRNAs targeting POMC 3′UTR
Source: Front Cell Neurosci. 2015 May 6;9:172. doi: 10.3389/fncel.2015.00172 (PMC4422035; doi:10.3389/fncel.2015.00172)
Supplement: Supplementary file 1 [file DataSheet1.DOC]

**Supplementary material**

**Western Blotting**

The hypothalami were collected 45 min and 30 min after intraperitoneal and intracerebroventricular injection, respectively. Tissue lysates were preparated by mashing in cold lysis buffer (50 mM Tris pH 7.9, 150 mM NaCl, 5 mM EDTA, 25 mM NaF, 1% Igepal, 0.5% Na deoxycholate, 0.1% SDS, 1 mM DTT, 2mM Na_3_VO_4_, 6.4 mg/ml PNPP, Protease Inhibitor Cocktail (*Sigma-Aldrich*)). A sample of lysate (40 µg) was boiled in 30 µl of Laemmli 2x Concentrate Sample Buffer (*Sigma-Aldrich*) for 5 minutes and then cooled in ice. 40 µg of total protein were separated by 15 % SDS-polyacrylamide gel electrophoresis and transferred to polyvinylidene fluoride membranes (450 mA, 105 minutes). Membranes were blocked with 10% fat-free milk in Tris-buffered saline containing 0.1% Tween-20 (TBST) buffer (50 mM Tris-HCl, pH 7.5, 138mM NaCl, and 0.1 % Tween 20) and were subsequently incubated with the following primary rabbit anti-Murine Leptin antibody 1:500 (*Abcam*) and this antibody was incubated at 4 °C overnight. Then, the membranes were washed with TBST and incubated for 2 hours with horseradish peroxidase-conjugated secondary anti-rabbit antibody (1:10000, *Jackson Immunoresearch*). Band were developped on membrane using the 1-Step Ultra TMB-Blotting Solution (*Thermo Scientific*).
