# Supplementary material for: Leptin is required for hypothalamic regulation of miRNAs targeting POMC 3′UTR
Source: Front Cell Neurosci. 2015 May 6;9:172. doi: 10.3389/fncel.2015.00172 (PMC4422035; doi:10.3389/fncel.2015.00172)
Supplement: Supplementary file 2 [file Image1.PDF]

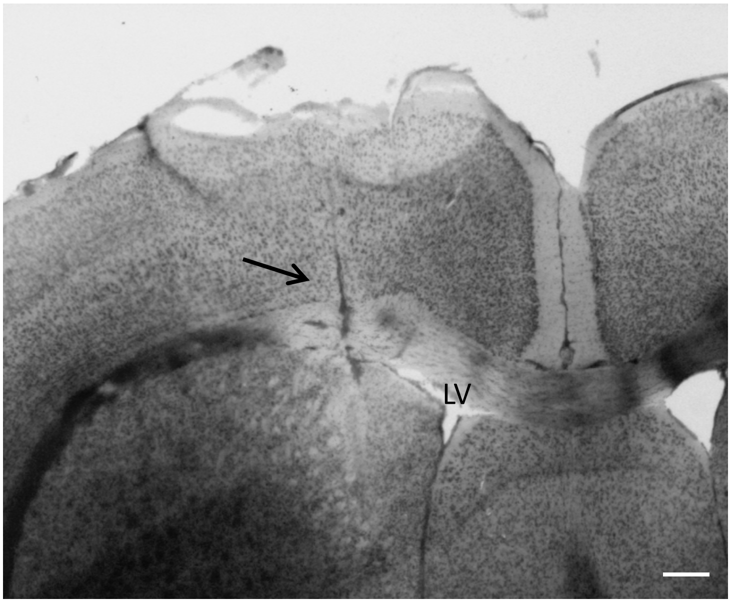

Supplemental Fig. 1: Representative section of the mouse brain showing the site of the injection at the lateral ventricle level. The arrow shows the tract of the cannula ending in the lateral ventricle. Scale bar = 500 µm
