# Supplementary material for: Leptin is required for hypothalamic regulation of miRNAs targeting POMC 3′UTR
Source: Front Cell Neurosci. 2015 May 6;9:172. doi: 10.3389/fncel.2015.00172 (PMC4422035; doi:10.3389/fncel.2015.00172)
Supplement: Supplementary file 3 [file Image2.PDF]

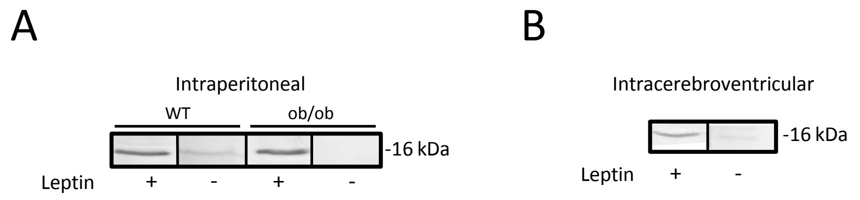

Supplemental Fig. 2: Leptin transport into the hypothalamus of intraperitoneal and intracerebroventricular treated animals.

Representative western blots of leptin protein level in hypothalamus of WT and ob/ob mice after peripheral (A) and central (B) administration of leptin.
