# Supplementary material for: Leptin is required for hypothalamic regulation of miRNAs targeting POMC 3′UTR
Source: Front Cell Neurosci. 2015 May 6;9:172. doi: 10.3389/fncel.2015.00172 (PMC4422035; doi:10.3389/fncel.2015.00172)
Supplement: Supplementary file 4 [file Image3.PDF]

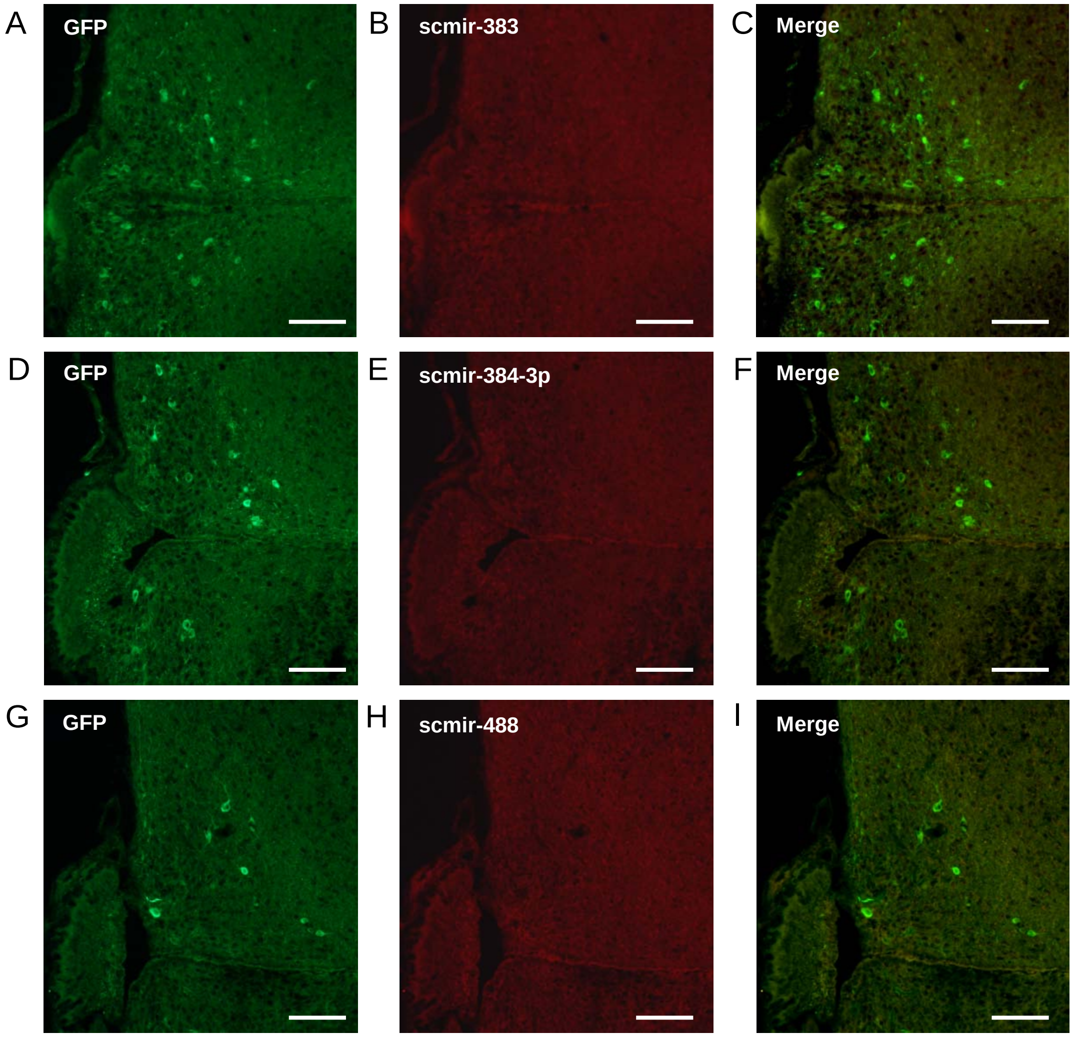

Supplemental Fig.3 . Negative Control using oligonucleotide scramble probes scmir-383, scmir-384-3p and scmir-488

Microphotography illustrating *in situ* hybridization and immunofluorescence experiments using oligonucleotide scramble probes in arcuate nucleus POMC-GFP mice. Scale bar = 50 µm.
